# Operando Characterization and Theoretical Modeling of Metal|Electrolyte Interphase Growth Kinetics in Solid-State Batteries. Part II: Modeling

**DOI:** 10.1021/acs.chemmater.2c03131

**Published:** 2023-01-28

**Authors:** Nicholas J. Williams, Edouard Quérel, Ieuan D. Seymour, Stephen J. Skinner, Ainara Aguadero

**Affiliations:** † Department of Materials, Imperial College London, Exhibition Road, LondonSW7 2AZ, U.K.; ‡ Department of Chemical Engineering, Massachusetts Institute of Technology, Cambridge, Massachusetts02139, United States; ¶ Instituto de Ciencia de Materiales de Madrid, ICMM-CSIC, Sor Juana Ines de La Cruz 3, 28049Madrid, Spain

## Abstract

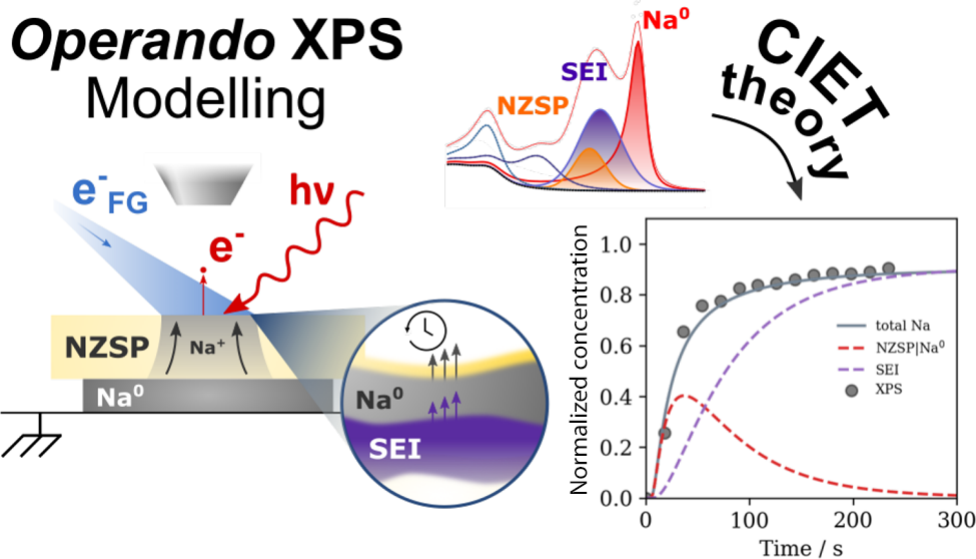

Understanding the interfacial dynamics of batteries is crucial to control degradation and increase electrochemical performance and cycling life. If the chemical potential of a negative electrode material lies outside of the stability window of an electrolyte (either solid or liquid), a decomposition layer (interphase) will form at the interface. To better understand and control degradation at interfaces in batteries, theoretical models describing the rate of formation of these interphases are required. This study focuses on the growth kinetics of the interphase forming between solid electrolytes and metallic negative electrodes in solid-state batteries. More specifically, we demonstrate that the rate of interphase formation and metal plating during charge can be accurately described by adapting the theory of coupled ion-electron transfer (CIET). The model is validated by fitting experimental data presented in the first part of this study. The data was collected operando as a Na metal layer was plated on top of a NaSICON solid electrolyte (Na_3.4_Zr_2_Si_2.4_P_0.6_O_12_ or NZSP) inside an XPS chamber. This study highlights the depth of information which can be extracted from this single operando experiment and is widely applicable to other solid-state electrolyte systems.

## Introduction

By virtue of their high capacities and low redox potentials, alkali metals constitute a class of negative electrode materials which could provide a step increase in the energy density of future generations of cells. Solid electrolytes (SEs) are employed in solid-state battery (SSB) cell types to enable the use of alkali metal negative electrodes.^1−5^ Yet, the high chemical potential of alkali metals make most SEs unstable against them.^1^ If the alkali metal | SE interface is unstable, a decomposition layer called the “interphase” is formed.^4−9^ Information about the interphase chemical composition and rate of formation are challenging to obtain because the reaction occurs at a buried interface. Understanding the decomposition reaction as it progresses would be extremely beneficial to then control it and limit its impact on the power performance and longevity of cells.^3^

**Figure 1:**
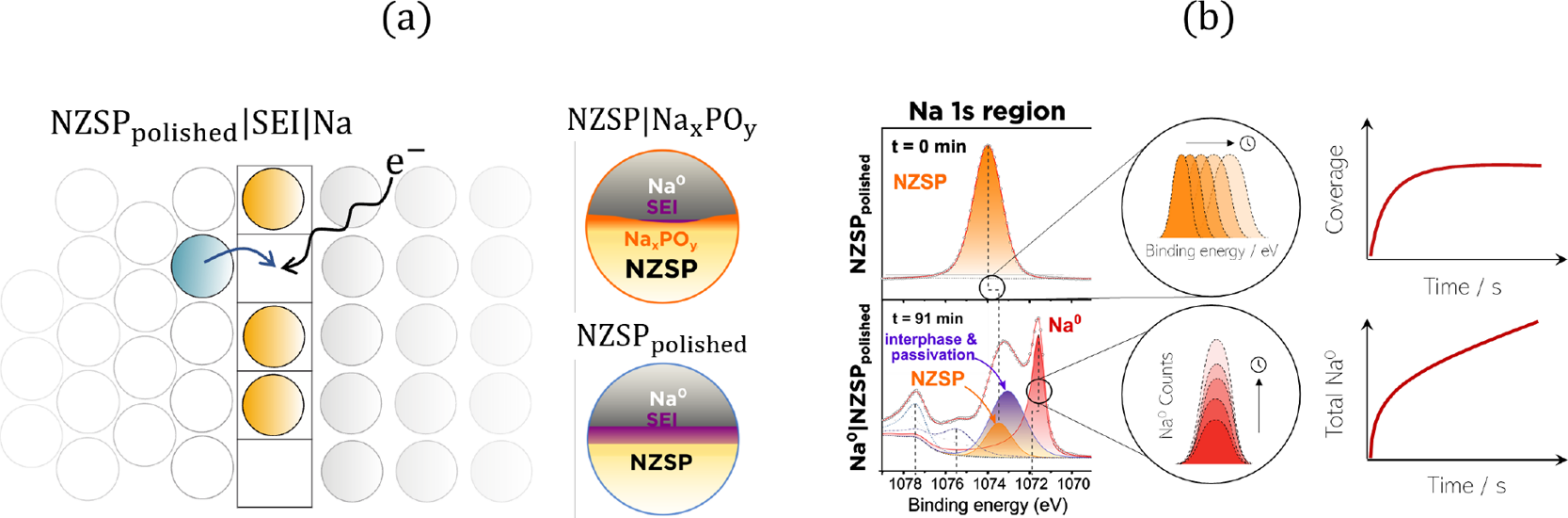
(a) Illustration of the electrode plating mechanism. When a resistive SEI is formed, the process has an entropic dependence where the probability of the reaction event occurring decreases as the NZSP surface becomes filled by the blocking SEI interphase. (b) Schematic illustration of the operando electroplating XPS spectra for the NZSP surface. The change in photoelectron binding energy is correlated with the coverage of a given interface on the NZSP surface, while the integrated area of the Na^0^ peak is correlated with the total amount of Na^0^ plated. XPS spectra are taken from Part I.^11^

The first part of this study described an XPS experiment which can be conducted to characterize the formation of an interphase between an SE and a layer of alkali metal. More specifically, this first article investigated the interphase forming between a NaSICON ceramic electrolyte (Na_3.4_Zr_2_Si_2.4_P_0.6_O_12_ or NZSP) and Na metal (Na^0^) as a model system.^10^ To analyze the formation of the interphase operando, the Na^0^|NZSP interface needs to be formed inside the XPS chamber.^5^ Besides, since XPS has a very limited depth of analysis, the thickness of the Na^0^ layer needs to be very thin to allow photoelectrons from deeper layers to escape to the surface. To overcome these issues, the Na^0^ layer was plated to the NZSP surface inside the XPS chamber from a counter electrode using low-energy electrons from the flood gun source as a “virtual electrode”. The electroplating event at the NZSP surface is given simply as (Figure 1a)
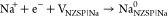
1where Na^+^, e^–^, *V*_NZSP|Na_, and Na_NZSP|Na_^0^ represent sodium ions, mobile electrons, vacancies on the NZSP surface, and sodium metal, respectively. This concerted reaction incorporates the transfer of ions from the electrolyte and electrons from the reservoir (flood gun in this case). It was established in a previous study^11^ that a solid electrolyte interphase (SEI, a type of interphase where the decomposition species are electronically insulating) forms upon plating at the interface between NZSP and Na^0^ (illustrated in Figure S1).

Interestingly, experimental work in Part I established that if the NZSP is covered by a thin Na_*x*_PO_*y*_ layer (natively present on the surface of as-sintered NZSP pellets), the decomposition of NZSP against Na metal can be prevented in great part.^11^ This was attributed to the protecting role of the Na_*x*_PO_*y*_ layer which is stable against Na metal.^6^ In terms of nomenclature, a distinction will be made between as-sintered NZSP samples terminated by a Na_*x*_PO_*y*_ layer (referred to as Na_*x*_PO_*y*_|NZSP) and polished NZSP samples (which do not have the Na_*x*_PO_*y*_ termination) referred to as NZSP_polished_.

This second part focuses on understanding how the kinetics of SEI formation and Na^0^ electroplating are interrelated in the operando XPS experiment. More specifically, the coupled ion-electron transfer (CIET) model is adapted to describe how Na^0^ plating on a NZSP surface is affected when a blocking SEI layer is formed at the Na^0^|NZSP interface. The simulated model is employed to fit XPS data from ref (11). The CIET model is able to accurately describe the evolution of the XPS peak areas and peak shifts as a function of plating time (Figure 1b). In particular, our work demonstrates that the binding energy shifts and broadening of peaks observed in the experimental data are correlated with the NZSP surface coverage, which is a variable in the model. The model also explains the evolution of the Na metal plating rate as a function of surface coverage. This second part of the study provides information about the kinetics of SEI formation and demonstrates the depth of information which can be extracted from a single XPS experiment to study the stability of a metal|SE interface.

## Theory

**Figure 2:**
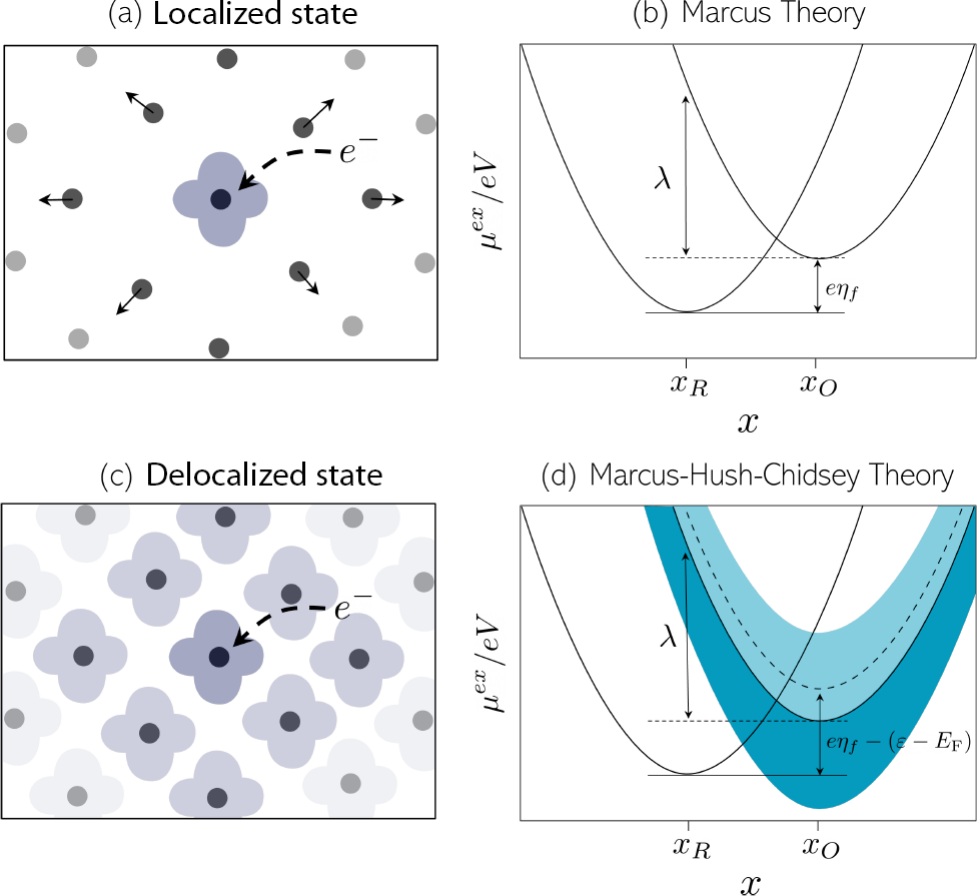
(a) Schematic illustration of an electron transfer event forming a localized electronic state where the arrows show environmental polarization of the local structure. (b) Excess chemical potential curves for the electron transfer as functions of the collective solvent coordinate (*x*) which corresponds to a nonadiabatic electron transfer event between two states via Marcus theory. (c) Schematic illustration of an electron transfer event forming a delocalized electronic state. (d) System where a metallic electron gains an additional electron as a result of the reduction reaction via Marcus–Hush–Chidsey theory. The parabola on the left corresponds to the reduced state, while the continuum on the right corresponds to the oxidized state where an additional electron occupies one of the one-electron states in the conduction band of the metal. The electron tunnelling event occurs at the intersection between the curves of the reduced and oxidized state, and the free energy of each reaction is the formal overpotential [plus the separation between the Fermi level (*E*_F_) and the unoccupied state the electron fills (ε) for the Marcus–Hush–Chidsey process].

The (electro)chemical potential, μ_*i*_ (eV), of a mobile species in an electrochemical system is expressed as^12−17^
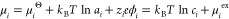
2where μ_*i*_^o^, *a*_*i*_, and ϕ_*i*_ represent the standard chemical potential, activity, and electrostatic potential of species *i*. The activity (*a*_*i*_ = γ_*i*_*c*_*i*_) is the product of the concentration and activity coefficient, which is a measure of the nonideality of the (electro)chemical potential using the excess chemical potential (μ_*i*_^ex^) which collects all nonidealities of species *i*.^13,15^ Here, we use the definition of (electro)chemical potential, meaning that if the species of interest is charged, we find the electrochemical potential, and if the species of interest is neutral, we find the chemical potential. At the electrode–electrolyte interface, the activation overpotential (η) describes the nonequilibrium shift in electrostatic potential between the electrons and ions for the general reduction reaction O^*n*+^ + *ne*^–^ → R as
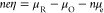
3where μ_R_, μ_O_, and μ_e_ represent the chemical potential of the reduced species, oxidized species, and free electron, and *n* represents the number of electrons transferred in the Faradaic reaction. The rate of electrochemical reactions is often described by the phenomenological Butler–Volmer (BV) equation, which was originally derived based on transition state theory to model the rate of ion transfer (IT).^13−15,18−20^ Here, the rate of ion migration over an activation barrier is determined through classical statistical thermodynamics devised of an attempt frequency and a success probability determined by the thermal energy of the system. Marcus theory explicitly described electron transfer (ET) as a tunnelling event which occurs when the reduced and oxidized states are iso-energetic (Figure 2a).^21,22^ By treating the electronic charge as a quantum particle, the activation energy barrier is determined by the dielectric polarization of the solvent environment (Figure 2b).^23,24^ In the case when an electron occupies a delocalized state in the conduction band of a metal as a result of the ET event, we adopt the Marcus–Hush–Chidsey theory (Figure 2c). In such a case, the rate for the ET event is obtained by integrating over all energy levels (ε) in the electrode
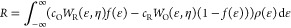
4where *c*_O_, *c*_R_, *W*_R_, *W*_O_, *f*, and ρ(ε) represent the concentration of oxidized species, concentration of reduced species, electron transfer probability for the forward reaction, electron transfer probability for the backward reaction, Fermi distribution function, and the density of electronic states in the electrode.^14,25,26^ In a recent study, Fraggedakis et al. developed the CIET model which treats ions using the classical transition state theory description and electrons using the quantum particle description.^14^ CIET then was used to accurately predict the rate of Li ion intercalation into LiFePO_4_ as a function of lithium concentration.^14^ CIET theory was later used to model the rate of Li ion intercalation and plating on a graphite particle.^27^ CIET theory can describe the rate of electrode plating, where one site in the transition state is excluded and where electrons are provided by a metallic phase^14,18,27−29^
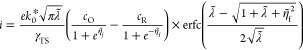
5where *k*_0_^*^ represents the rate constant which includes ion transfer, thermal activation, and the electron tunnelling probability;  is the reorganization energy; and  is the formal overpotential. When the reaction is not limited by the rate of ion transfer, γ_TS_ = 1. The reduced species may fill a RedOx site, causing the current to change as a function of filling fraction (*c*).

When considering the effects of SEI formation, which is thought to block the plating reaction (Figure 1), the rate constant has an entropic constraint and must be proportional to the availability of free reaction sites on the NZSP surface. Thus, the activity coefficient of the transition state (γ_TS_) for the electrode plating is given as^14,30^
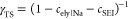
6where *c*_ely|Na_ represents the concentration of sodium metal in contact with the solid electrolyte (ely = NZSP for NZSP_polished_ or ely = Na_*x*_PO_*y*_ for NZSP| Na_*x*_PO_*y*_) and *c*_SEI_ represents the concentration of SEI material blocking the plating reaction. By combining eq 5 and 6 we can derive the concentration-dependent current density
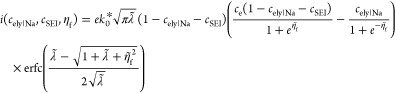
7where . The concentration of electrons, *c*_e_, at the surface of the electrolyte is determined by the external electron current. Details of the cell voltage and overpotential are given in the Supporting Information.

**Figure 3:**
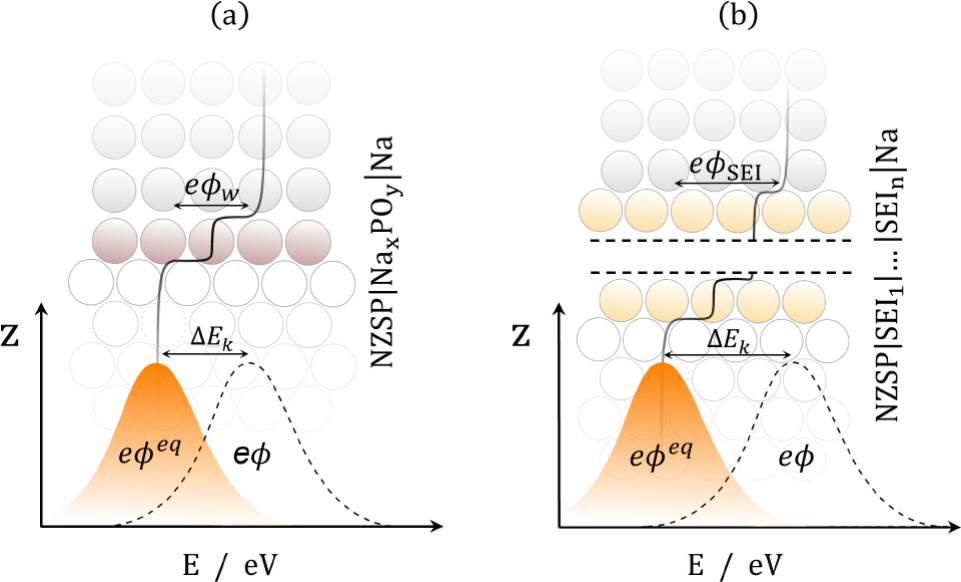
Schematic illustration of the formation of the interfacial voltages at the NZSP|Na interface for (a) the as-sintered sample where a Na_*x*_PO_*y*_ layer protects the NZSP surface from reacting with the Na phase, and for (b) the polished sample where we observe the formation of resistive SEI and many additional interfacial voltages. The XPS spectra indicate that the shift in photoelectron kinetic energy is equal to the interfacial voltage.

When an interface is formed between two materials, the change in the electrostatic potential (Δ*V*) is directly related to the change in the electron density (ρ) according to Poisson’s equation^31^
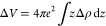
8Here, the electrostatic potential (*V*) is the difference between the vacuum level and the average of the electrostatic potential in the bulk of the material, the delta symbol (Δ) represents the difference between the states before and after the interface is formed, and *z* is the direction of the line integral.^32^ The change in electrostatic potential is illustrated in Figure 3, where as sodium plates, an interfacial voltage at the surface of the working electrode is formed (ϕ_*w*_). This can be derived from eq S7 in the Supporting Information
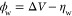
9where we can interpret ϕ_w_ as the applied potential at the working electrode.^27^ As a photoelectron leaves the NZSP phase, it will undergo acceleration in accordance with the magnitude of the interfacial voltage.^33,34^ The shift in photoelectron kinetic energy (*E*_k_) is therefore equivalent to the interfacial voltage^35,36^
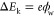
10

The experimentally observed shift in kinetic energy of the photoelectron  is spatially averaged and is therefore proportional to the concentration (*c*) of the newly formed interface
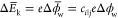
11where we assume that the initial concentration of material at the surface is negligible and that the structure of the metallic phase formed on the surface is thin and does not screen electrostatic charge. For the NZSP_polished_ system, we also observe the formation of new SEIs, resulting in an additional interfacial voltage term (ϕ_SEI_) which is derived in eq S8. The SEI will impose additional acceleration on the photoelectron leaving the NZSP phase due to the interfacial voltages which forms at the NZSP|SEI and SEI|Na interfaces, plus any interfaces which form between all of the SEIs themselves (Figure 3). The magnitude of the summed interfacial voltages is expected to be large relative to the as-sintered sample due to significant changes in the electron density (eq 8) as a result of decomposition. The spatially averaged shift in photoelectron kinetic energy is given as a function of concentration of each interface and is therefore given as
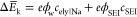
12where
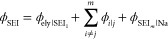
13where *m* represents the total number of SEI interfaces between the NZSP and Na phases and *i* and *j* represent the phases in contact.

## Results and Discussion

**Figure 4:**
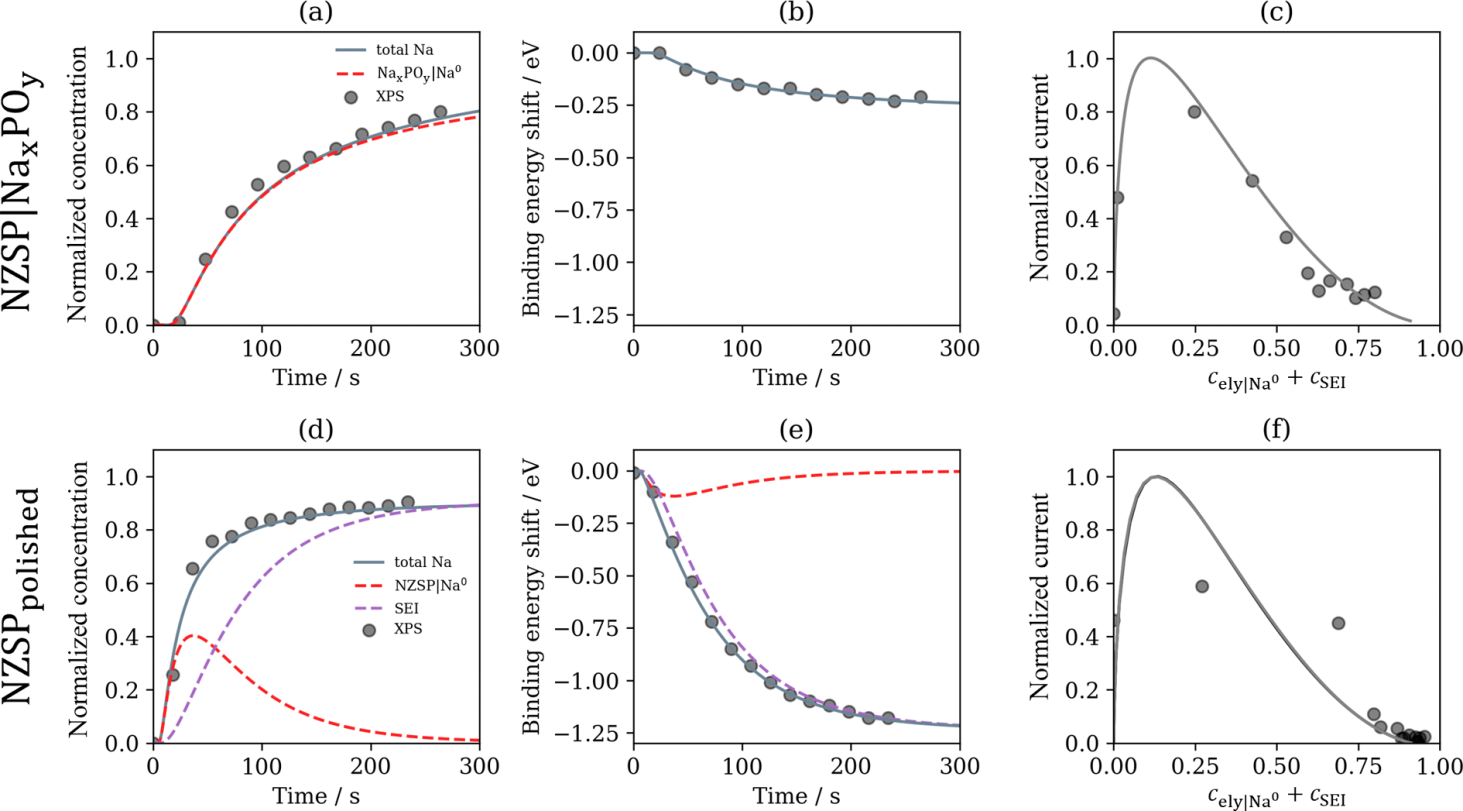
(a and d) Normalized concentration surface species as a function of time, (b and e) normalized electron binding energy shift as a function of time, and (c and f) normalized plating current as a function of surface coverage for the NZSP| Na_*x*_PO_*y*_ and NZSP_polished_ systems, respectively. Dots are the experimental data, and the lines are the fits from the simulation.

To determine effects of Na_*x*_PO_*y*_ and SEI interphases on the rate of electrode plating, we simulated the plating mechanism for both systems (details of the simulation are given in the Supporting Information). For NZSP|Na_*x*_PO_*y*_, where the formation of an insulating SEI is not spontaneous, eq 7 fits the experimental data by optimizing the concentration normalization (*c*_max_), reorganization energy , and overpotential of the working electrode  (Figure 4a,b). The apparent reorganization energy derived here is comparable to lithium ion intercalation into LiFePO_4_ and graphite .^27,30^

The solid gray and dashed red lines in Figure 4a represent the total sodium plated and the concentration of the Na_*x*_PO_*y*_|Na interface, respectively. By extending the simulation time beyond the experimental observation, we calculate that the plating reaches a steady state when  (Figure S3). By analyzing the shift in photoelectron binding energy, we calculated the interfacial voltage to be . The agreement between the sodium metal concentration (Figure 4a) and interfacial concentration (Figure 4b) supports our theory that within the limits of the plating process, diffusion of sodium metal away from the reaction site is slow, and we therefore believe that a monolayer structure is forming on the surface. Figure 4c illustrates the observed sodium plating current (*i* = *e∂*_*t*_*c*) as a function of sodium concentration. The autocatalytic nature of the plating process means the CIET reaction is slow at the beginning of the experiment when the concentration of sodium metal is low.^14,29,37^ The plating process is autocatalytic at low concentration, as redox-active molecules increase the exchange rate for electron transfer.^37^ Therefore, once plating initiates we observe a sharp increase in the plating rate, which we predict to peak at a surface concentration of approximately *c*_ely|Na_ = 0.1. Plating is autoinhibitory at high concentrations, as product covers the active sites. Beyond a critical concentration, the plating process exponentially decreases in rate as surface vacancies fill and autoinhibition is observed.^13,14^

Following eq 9, the shift in electrostatic potential can be approximated by adding the overpotential  to the interfacial voltage  to yield Δ*V* = −0.39*V*. The interfacial voltage can also be derived from the Poisson equation (eq 8) whereby electrons are transferred across the interface, leading to the creation of an interfacial dipole, which induces a step in the electrostatic potential at the interface.^15,31,34^

For the NZSP_polished_ system, we consider the concentration of the SEI interface. The specific interfacial concentrations are not experimentally observable due to attenuation of the photoelectrons and must therefore be approximated via simulation of the parallel plating and decomposition processes (Figure S1). Figure 4d illustrates plating on the NZSP surface (dashed red line) followed by the formation of an insulating SEI (dashed purple line). The reorganization energy  and overpotential of the working electrode  agree well with the NZSP| Na_*x*_PO_*y*_ system.

We postulated that electrode plating occurs prior to SEI formation; thus, the initial stage of sodium formation is relatively unimpeded. However, the plating reaction is slowed as the unstable NZSP|Na interface undergoes decomposition into SEI products.^6^ Upon plotting the plating rate against the NZSP surface coverage (*c*_ely|Na_ + *c*_SEI_), we were able to fit the experimental data in Figure 4f. By extending the simulation time beyond the experimental observation, we calculate that the plating rate approaches zero as the surface is almost completely filled by the blocking SEI (Figure S3). We were able to plot the rate of CIET explicitly as a function of *c*_ely|Na_ and *c*_SEI_ as the filled red line on the contour plot (Figure S2). Moreover, the theory of an insulting SEI formation on the NZSP_polished_ surface agrees with previous EIS data, which suggests that the electrode plating is rate-limiting for the polished sample where insulating SEI products block ion and electron transfer.^6^ We note that the order in which plating and SEI formation occurs means that the SEI formation process is spontaneous upon the formation of the electrolyte|Na interface. In a conventional battery device, SEI formation will therefore occur as soon as the electrolyte makes contact with metal anode, and we will not expect to see the same drop in performance upon the first cycle as observed in this experiment. For a zero-excess metal anode setup, this experiment is representative of the first cycle of the real system where the same kinetics effects are likely be observed. This experimental setup is not confined to studying the solid electrolyte–electrode interface, where electroplating of sodium on a current collector (e.g., in a “zero excess capacity” negative electrode cell configuration) could also be investigated.

Analysis of Figure 4e shows that ϕ_w,NZSP|Na_ = −0.30 V and ϕ_SEI_ = −1.36 V. The shift in electrostatic potential at the NZSP|Na interface (eq 9) is approximated as Δ*V* = −0.39 V, which is well aligned with that of the Na_*x*_PO_*y*_|Na interface. This suggests that prior to decomposition, the interface between the ceramic and metal phases has a similar electronic structure. The large shift in interfacial voltage caused by the SEI is accounted for by the significant change in electron density as a result of interfacial decomposition. We can therefore conclude that no significant decomposition processes are occurring at the Na_*x*_PO_*y*_|Na^0^ due to its relatively small shift in photoelectron binding energy.

The analysis procedure given here allows us to directly observe the effects of the autocatalysis and autoinhibitory on the rate of electrode plating. Moreover, it may be possible to predict the presence of SEI formation by combining the shift in kinetic energy of the photoelectron and the concentration of material plated onto the surface of the electrolyte. Beyond studying the kinetics of SEI growth, this model allows us to characterize the SE surface coverage and study its kinetics. Surface coverage is important to control, for instance, in the first plating cycle of cells with a “zero excess capacity” anode. This experimental protocol and associated theoretical models offer a solution to study the evolution of surface coverage operando and compare the performance of different SE (or interlayer) surfaces. This analysis technique has potential to characterize the formation of any electrochemical interface where electrostatic screening is negligible, such as degradation of Li–air batteries, nitrogen reduction, and fuel cell systems.

The time-evolved thickness of the buried SEI is difficult to analyze experimentally and of particular interest to the battery community. The model proposed in this study assumes the SEI is simply a RedOx blocking layer, and the time-evolved nature of the SEI is not considered. In a further study, the ordinary differential equation constrained optimization model could be adapted to a partial differential equation constrained optimization model to take account for the thickness, diffusivity, and RedOx activity of the SEI.

## Conclusion

We have combined operando X-ray photoelectron spectroscopy and coupled ion-electron transfer theory to advance our understanding of the effects of Na_*x*_PO_*y*_ and SEI interfaces on sodium plating at the NZSP surface. Using the integrated Na^0^ peak as the reference for reaction extent, we were able to interpret the rate of plating as functions of sodium metal concentration and validate the use of CIET theory to model the rate of plating.^14^ For the NZSP|Na_*x*_PO_*y*_ system, we observe the effects of autocatalysis and autoinhibition caused by the sodium metal on the solid electrolyte surface. The modeling suggests that no blocking SEI is formed on the Na_*x*_PO_*y*_ surface. On the other hand, we observed that the rate of plating on the NZSP_polished_ system is impeded by the decomposition of the NZSP|Na interface. We introduced a blocking SEI layer to the CIET equation, where the decrease in rate is proportional to the concentration of SEI. This constraint allowed us to approximate the concentration of the NZSP|Na and NZSP|SEI interfaces over the course of the plating process. Using the shifts in photoelectron kinetics energy, we validated the simulation results and determined that a blocking SEI phase is responsible for the vanishing electrode plating rate.

## Computational Methods

To carry out the ODE constrained optimization we used both the integrated Na^0^ peak and the binding energy shift data simultaneously. Details of the kinetics model can be found in the Supporting Information.

## Data Availability

The XPS data and fitting models used in this work are accessible on the following GitHub repository: https://github.com/nw7g14/Modelling-XPS-ODE-constrained-opt.

## Supporting Information Available

Figures S1–S3 and supplementary notes: derivations of the chemical potential, cell voltage, kinetic model, activation overpotential, and long time scale simulation data (PDF)
