# Supplementary material for: Operando Characterization and Theoretical Modeling of Metal|Electrolyte Interphase Growth Kinetics in Solid-State Batteries. Part II: Modeling
Source: Chem Mater. 2023 Jan 28;35(3):863–9. doi: 10.1021/acs.chemmater.2c03131 (PMC9933423; doi:10.1021/acs.chemmater.2c03131)
Supplement: Supplementary file 1 — cm2c03131_si_001.pdf [file cm2c03131_si_001.pdf]

Operando characterization and theoretical modelling of metal|electrolyte interphase growth kinetics in solid-state-batteries - Part II: Modelling

Nicholas J. Williams,[*,†,‡] Edouard Quérel,[†] Ieuan D. Seymour,[†] Stephen J. Skinner,[†] and Ainara Aguadero[¶,†]

†*Department of Materials, Imperial College London, Exhibition Road, London SW7 2AZ, UK*

‡*Department of Chemical Engineering, Massachusetts Institute of Technology, Cambridge, MA, 02139, USA*

¶*Instituto de Ciencia de Materiales de Madrid, ICMM-CSIC, Sor Juana Ines de La Cruz 3, 28049, Madrid, Spain*

E-mail: n.williams18@imperial.ac.uk

Supplementary Information

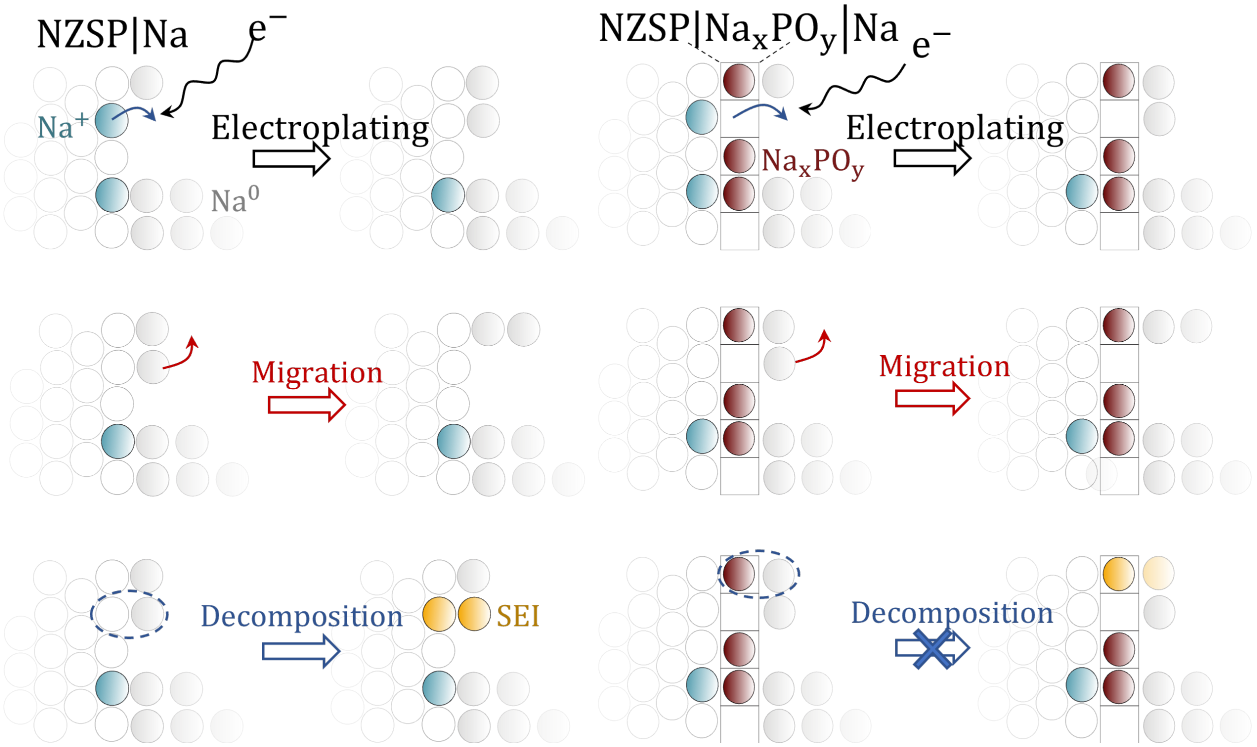

Figure S1: Schematic illustration of the electroplating, migration and decomposition reactions for the polished (left) and as-sintered (right) systems, where blue, grey, red and yellow spheres represent sodium ions in NZSP, plated sodium metal, sodium phosphate interface and blocking SEI interphase, respectively.

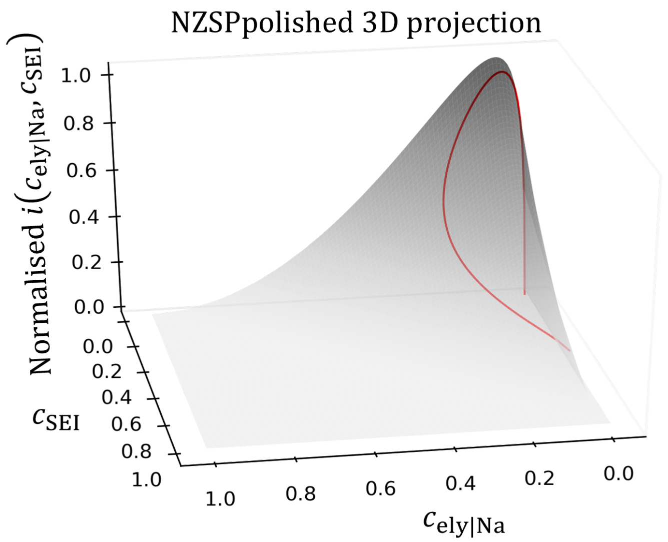

Figure S2: Normalised sodium plating current as a function of sodium metal coverage and SEI coverage. The red line tracks the trajectory of the reaction observed experimentally.

Chemical Potential and Cell Voltage

The experimental setup means there is no potentiostat to determine the cell voltage at open circuit or equilibrium. Using chemical potentials and nonequilibrium thermodynamics we will derive the cell voltage. The affinity (A_{res}) describes the direction in which a reduction reaction will progress:[1]

$$A_{res} = ne\eta \tag{S1}$$

The affinity can also be expressed as the difference between the externally controlled potential (μ_{res}) and the internal energy (μ_h):[1–3]

$$\mu_{res} = A_{res} + \mu_h \tag{S2}$$

The internal energy is the difference in chemical potential between the reference (bottom) and working (top) electrodes.

NZSPAS: The electrode-electrolyte interface is stable, the electrode plating (working electrode) and electrode stripping (reference electrode) reactions can be described as:

$$\mathrm{Na}_w^+ + \mathrm{e}_{FG}^- + \mathrm{V}_{\mathrm{Na_xPO_y|Na}} \rightarrow \mathrm{Na}^0_{\mathrm{Na_xPO_y|Na}} \tag{S3}$$

$$\mathrm{Na}_r^0 \rightarrow \mathrm{Na}_r^+ + \mathrm{e}_r^- \tag{S4}$$

where $\mathrm{Na}^0_{\mathrm{Na_xPO_y|Na}}$ and $\mathrm{V}_{\mathrm{Na_xPO_y|Na}}$ represent sodium metal and sodium metal vacancies at the Na_3PO_4 surface of the working electrode, and e_{FG}^- represents the electron coming from the floodgun. The stripping reaction in eq. S4 does not include details of the electrode-electrolyte interface as we assume the chemical potential of the interface is invariant. Using eq. 5, the overpotential at the working (η_w) and reference (η_r) electrodes can be derived as:

$$e\eta_w = \mu_{\mathrm{Na}^0_{\mathrm{ely|Na}}} - \mu_{\mathrm{V}_{\mathrm{ely|Na}}} - \mu_{\mathrm{Na}^+_w} - \mu_{\mathrm{e}^-_w} \tag{S5}$$

$$e\eta_r = \mu_{\mathrm{Na}^0{}_r} - \mu_{\mathrm{Na}^+_r} - \mu_{\mathrm{e}^-_r} \tag{S6}$$

where the internal energy describes the difference in chemical potential of each electrode $\mu = \mu_{\mathrm{Na}^0_{\mathrm{ely|Na}}} - \mu_{\mathrm{V}_{\mathrm{ely|Na}}} - \mu_{\mathrm{Na}^0{}_r}$ and is the binding energy of Na metal to the electrolyte surface.[4] We assume fast Na ion diffusion and therefore the gradient in electrostatic and chemical potential in the electrolyte is negligible, as such $\mu_{\mathrm{Na}^+_w} \approx \mu_{\mathrm{Na}^+_r}$. Using sodium metal as the reference potential $\mu^\Theta_{\mathrm{Na}^0_r} = \mu^\Theta_{\mathrm{Na}^0_w} \approx 0$, the reservoir chemical potential is given as:

$$\mu_{res} = \mu - e\eta_w = e\phi_w \tag{S7}$$

where we assume the reaction at the reference electrode is facile, thus $\eta_r \approx 0$.[4] The cell voltage, $V = -\mu_{res}/ne$, therefore has electrostatic and chemical contributions.

NZSPpolished: The electrode plating reaction now occurs directly on the NZSP surface:

$$\mathrm{Na}^+_w + \mathrm{e}^-_w + \mathrm{V}_{\mathrm{NZSP|Na}} \rightarrow \mathrm{Na}^0_{\mathrm{NZSP|Na}} \tag{S8}$$

where $\mathrm{Na}^0_{\mathrm{NZSP|Na}}$ and $\mathrm{V}_{\mathrm{NZSP|Na}}$ represent sodium metal and sodium metal vacancies at the NZSP surface of the working electrode. The electrode stripping reaction is consistent with eq. S4. The overpotential at the working electrode has the same derivation as for the as-sintered sample (eq. S10), where the chemical potential of the homogeneous metallic phase is given as $\mu_h = \mu_{\mathrm{Na}^0_{\mathrm{NZSP|Na}}} - \mu_{\mathrm{V}_{\mathrm{NZSP|Na}}}$.

The SEI formation reaction $\mathrm{Na}^0_{\mathrm{NZSP|Na}} \rightarrow \mathrm{SEI}$ can be expanded using eq. S8:

$$\mathrm{Na}^+_w + \mathrm{e}^-_w + \mathrm{V}_{\mathrm{NZSP|Na}} \rightarrow \mathrm{SEI} \tag{S9}$$

This yields the chemical potential for formation of the SEI phase, μ_{SEI}. The cell volt-

age will therefore depend on the rate at which each reaction (plating or decomposition) is occurring:

$$\mu_{res} = e\eta_w + \mu_{\text{SEI}} + \mu \tag{S10}$$

Since there is no contribution to the cell voltage from the reference electrode, we find the reservoir potential is equal to the potential of the working electrode:

$$\mu_{res} = e\phi_w \tag{S11}$$

Kinetic Model

To simulate the plating process, a system of nonlinear ordinary differential equations was derived.

NZSPAS: Two reactions are considered; R_1 is the CIET on the Na_3PO_4 surface, and R_2 is diffisuion of sodium metal away from the Na_3PO_4 surface.

$$R_1 = k_1^* \sqrt{\pi\tilde{\lambda}}(1 - c_{\text{ely}|\text{Na}}) \left(\frac{1 - c_{\text{ely}|\text{Na}}}{1 + e^{\tilde{\eta}_f}} - \frac{c_{\text{ely}|\text{Na}}}{1 + e^{-\tilde{\eta}_f}} \right) \times \text{erfc}\left(\frac{\tilde{\lambda} - \sqrt{1 + \tilde{\lambda} + \tilde{\eta}_f^2}}{2\sqrt{\tilde{\lambda}}} \right) \tag{S12}$$

$$R_2 = \overrightarrow{k}_2 c_{\text{ely}|\text{Na}} - \overleftarrow{k}_2 (1 - c_{\text{ely}|\text{Na}}) \tag{S13}$$

NZSPpolished Three reactions are concidered R_1 is the CIET on the $NZSP$ surface, R_2 is diffisuion of sodium metal away from the NZSP surface, and R_3 is the decomposistion of the NZSP|Na interface to from the blocking SEI.

$$R_1 = k_1^* \sqrt{\pi\tilde{\lambda}}(1 - c_{\text{ely|Na}} - c_{\text{SEI}}) \left(\frac{1 - c_{\text{ely|Na}} - c_{\text{SEI}}}{1 + e^{\tilde{\eta}_f}} - \frac{c_{\text{ely|Na}}}{1 + e^{-\tilde{\eta}_f}} \right) \times \text{erfc} \left(\frac{\tilde{\lambda} - \sqrt{1 + \tilde{\lambda} + \tilde{\eta}_f^2}}{2\sqrt{\tilde{\lambda}}} \right) \tag{S14}$$

$$R_2 = \overrightarrow{k}_2 c_{\text{ely|Na}} - \overleftarrow{k}_2 (1 - c_{\text{ely|Na}} - c_{\text{SEI}}) c_{\text{Na}^0} \tag{S15}$$

$$R_3 = \overrightarrow{k}_3 c_{\text{ely|Na}} - \overleftarrow{k}_3 c_{\text{SEI}} \tag{S16}$$

Activation Overpotential

The fitting of eq. 8 to the data in Fig. 3 assumes that the activation overpotential driving the electroplating process is constant. Here we will derive the origin of the activaiton overpotential. The change in nonequilibrium free energy ($\Delta G(c, i)$) of the sodium plating reaction as a function of concentration and current is given as:

$$\Delta G(c, i) = \Delta G(c, 0) + \Delta_i W_d(c, i) \tag{S17}$$

where $\Delta G(c, 0)$ is the change in equilibrium free energy at zero current and $\Delta_i W_d(c, i)$ is the irreversible driving work done on a system. The irreversible driving work done is given as the following time integral:[1]

$$\Delta_i W_d(c, i) = \int_{t_0}^{t} i^2 R_F \, dt \tag{S18}$$

where R_F is the resistance of the Faradic resistance, $R_F = -\eta/i$. The irreversible driving work done can also be given as the following concentration integral:[1]

$$\Delta_i W_d(c, i) = \int_{c_0}^{c} (\mu_{res} - \mu_h) \, dc \tag{S19}$$

By combing eq. S1 and S2 with S17, we assume that the overpotential is invariant over the concentration range and yield the solution:

$$\int_{c_0}^{c} (\mu_{res} - \mu_h)\, dc = -e\eta c \tag{S20}$$

The current was calculated as the rate of sodium metal plating, $i = e\partial_t c$, thus the time integral in eq. S18 and S20 can be combined to give:

$$-e\eta c = -e \int_{t_0}^{t} \eta \partial_t c\, dt \tag{S21}$$

If again we assume the overpotential to be invariant over the time range, then:

$$-e\eta c = -e\eta \int_{t_0}^{t} \partial_t c\, dt \tag{S22}$$

where the integral $\int_{t_0}^{t} \partial_t c\, dt = c$. Thus, the overpotential does not vary with concentration.

Simulation Data:

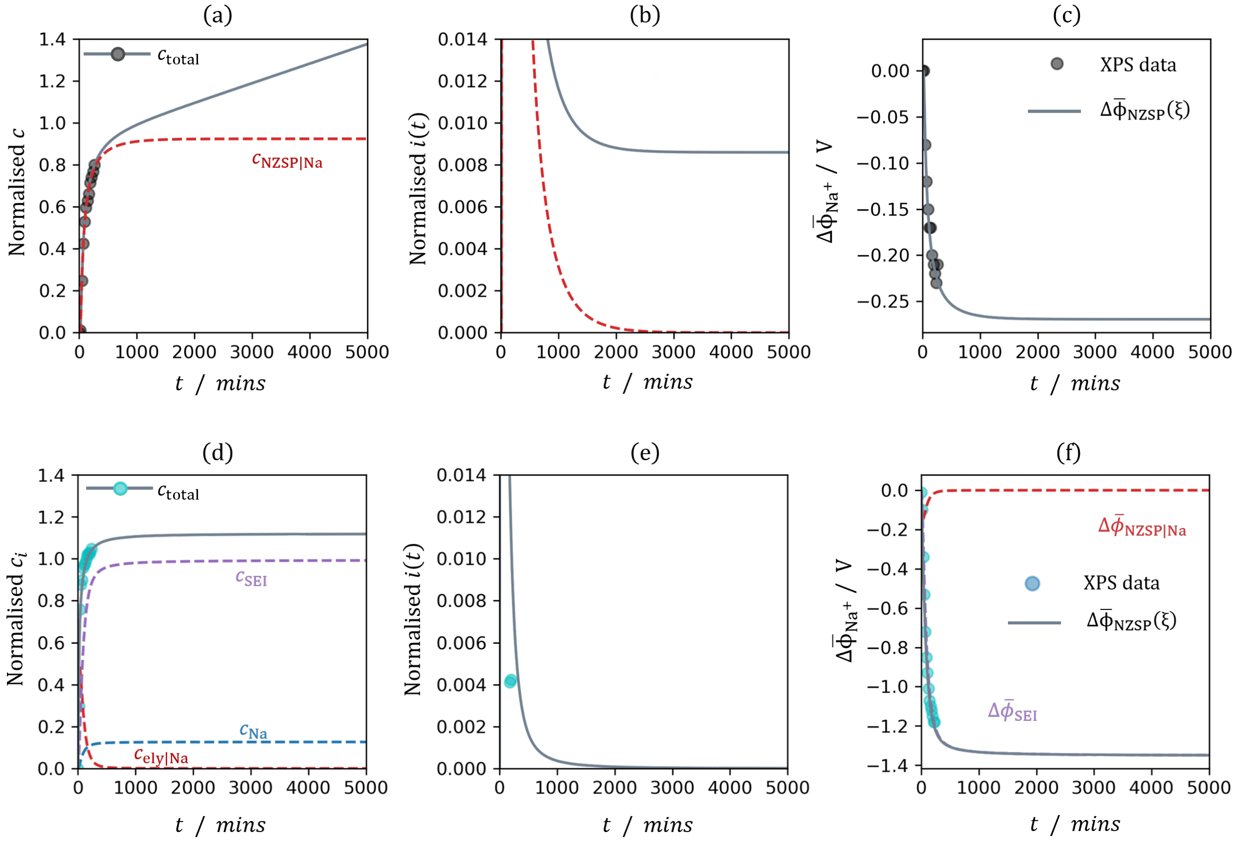

Figure S3:(a) Normalised concentration surface species, (b) Normalised rate of electrode plating, and (c) spatially averaged shift in sodium ion potential as a function of time for the as-sintered sample, where the dashed red line represent the simulated concentration of the Na_xPO_y|Na interface. (d) Normalised concentration surface species, (e) normalised rate of electrode plating, and (c) spatially averaged shift in sodium ion potential as a function of time for the polished sample, where the dashed purple and red lines represent the simulated concentration of the NZSP|SEI|Na and NZSP|Na interfaces, respectively. All circle points are taken from experimental data.

References

(1) Bazant, M. Z. Thermodynamic stability of driven open systems and control of phase separation by electro-autocatalysis. *royal society of chemistry* **2017**, *199*, 423–463.

(2) Rao, R.; Esposito, M. Nonequilibrium thermodynamics of chemical reaction networks: Wisdom from stochastic thermodynamics. *Physical Review X* **2016**, *6*, 1–23.

(3) Fraggedakis, D.; McEldrew, M.; Smith, R. B.; Krishnan, Y.; Zhang, Y.; Bai, P.; Chueh, W. C.; Shao-Horn, Y.; Bazant, M. Z. Theory of coupled ion-electron transfer kinetics. *Electrochimica Acta* **2021**, *367*, 137432.

(4) Gao, T.; Han, Y.; Fraggedakis, D.; Das, S.; Zhou, T.; Yeh, C. N.; Xu, S.; Chueh, W. C.; Li, J.; Bazant, M. Z. Interplay of Lithium Intercalation and Plating on a Single Graphite Particle. *Joule* **2021**, *5*, 393–414.
